# Brainstem Encephalitis Following COVID-19 Vaccination: A Case Report

**DOI:** 10.7759/cureus.104413

**Published:** 2026-02-27

**Authors:** Akihito Koseki, Youji Suzuki, Shugo Uchida, Naoki Morishita, Naoki Sakai

**Affiliations:** 1 Neurology, Yaizu City Hospital, Yaizu, JPN; 2 Neurology, Hamamatsu Medical Center, Hamamatsu, JPN

**Keywords:** adverse event, brainstem encephalitis, covid-19, covid-19 vaccines, mrna vaccines

## Abstract

Encephalitis is an extremely rare neurological adverse event following vaccination against coronavirus disease 2019 (COVID-19). We report a case of brainstem encephalitis following mRNA COVID-19 vaccination. A 30-year-old Japanese female presented with acute diplopia characterized by recurrent, paroxysmal episodes following the second dose of the vaccine, which resolved spontaneously. Brain magnetic resonance imaging revealed inflammatory changes in the brainstem. The patient responded rapidly to steroid therapy and subsequently improved. This case shares key clinical features with another case, including similarities in symptoms, lesion localization, and steroid responsiveness. However, it differs with respect to the timing of symptom onset and the presence of mild residual deficits. This report makes a contribution to the limited literature on this rare post-vaccination condition by documenting its clinical features.

## Introduction

The pandemic caused by severe acute respiratory syndrome coronavirus 2 (SARS-CoV-2) presented an unprecedented global public health crisis. In response, the rapid development and deployment of vaccines were crucial in controlling the pandemic. However, as vaccination programs expanded worldwide, various neurological adverse events, although rare, have been reported and recognized as clinically significant complications. Although neurological adverse events following vaccination are generally mild and transient, they may also cause serious complications, such as cerebral venous sinus thrombosis, Bell's palsy, acute transverse myelitis, acute disseminated encephalomyelitis, and acute demyelinating polyneuropathy [1].

Among these, encephalitis following coronavirus disease 2019 (COVID-19) vaccination is extremely rare. Large-scale epidemiological data identified 79 cases among 99.3 million administered doses of the mRNA COVID-19 vaccine ChAdOx1 nCov-19 (AZD1222) and 20 cases among 110.6 million doses of BNT162b2, corresponding to incidences of approximately 0.08 and 0.02 per 100,000 doses, respectively [2]. Furthermore, brainstem encephalitis was particularly rare, with only one case identified in a cohort study of 19 central nervous system disorders that occurred following COVID-19 vaccination [3]. Due to the paucity of reported cases, the clinical characteristics and optimal management strategies of brainstem encephalitis remain unclear. Accordingly, the accumulation of well-documented clinical data is essential. We herein report a case detailing the clinical course of brainstem encephalitis following COVID-19 vaccination to further advance understanding of this rare complication.

## Case presentation

**Figure 1:**
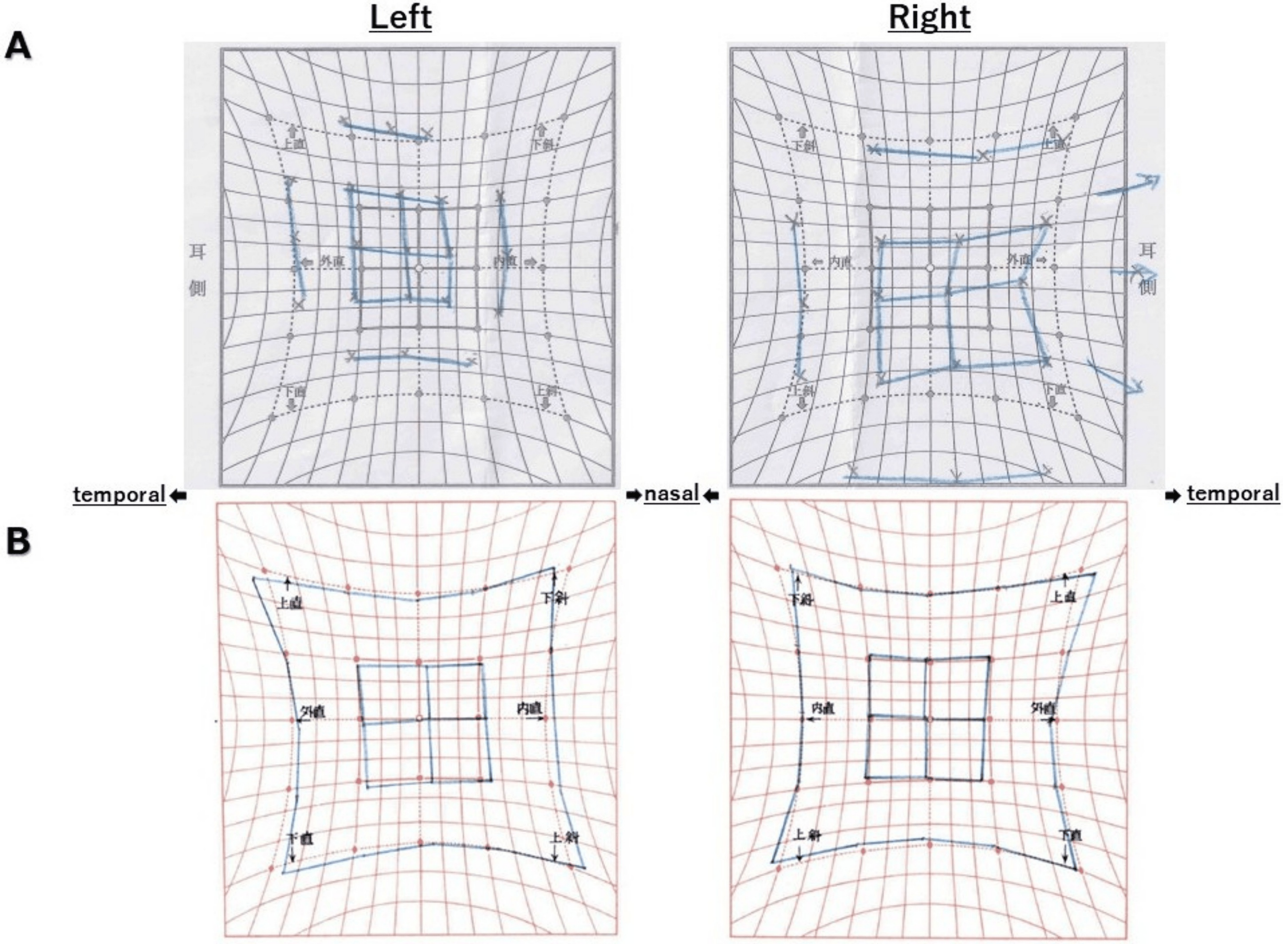
Hess chart. The left and right eyes are represented in the figures on the left and right, respectively. On day 60, the left eye demonstrated restricted adduction and infraduction, with compensatory excess abduction in the right eye (A). On day 61, slight abduction restriction was observed in the right eye, with compensatory excess adduction in the left eye (B). These oculomotor deficits are thought to be caused by brainstem lesions and are considered to be the cause of her diplopia.

A 30-year-old Japanese female presented with sudden-onset diplopia and bilateral ocular pain. She had a history of migraines and had undergone tonsillectomy for tonsillitis. She received the COVID-19 vaccine (BNT162b2) on days one and 22. After the first vaccination, she developed a fever of approximately 37°C, and after the second, she experienced malaise. Both conditions resolved within one day. Spontaneous pain in the back of both eyes developed a few days after the second vaccination. From day 43, she experienced diplopia one to two times daily, irrespective of gaze direction, including the primary position. The episodes were transient and resolved spontaneously. Around day 58, she developed numbness and hypoesthesia in the left buccal region that improved without treatment. On the morning of day 60, she complained of diplopia and visited an ophthalmologist, where examinations revealed adduction and infraduction deficits in the left eye and compensatory excessive abduction in the right eye (Figure 1). On the morning of day 61, her eye position normalized. However, bilateral ocular pain persisted, and she was admitted to our hospital.

On admission, her height and weight were 163 cm and 52 kg, and her vital signs were as follows: body temperature of 36.8°C, heart rate of 100 beats/min, blood pressure of 111/79 mmHg, respiratory rate of 12 breaths/min, and oxygen saturation of 97%. She was conscious and exhibited normal cognitive function. Her visual field was intact, and her pupils measured 4 mm bilaterally, with normal pupillary light reflex. No nystagmus was present. She experienced mild abduction limitation in the right eye and compensatory excessive adduction in the left eye (Figure 1). Bilateral eye pain was also noted, but no ptosis was observed. Other cranial nerve findings were normal. Muscle strength and deep tendon reflexes were intact, and pathological reflexes were absent. No abnormalities were detected in the sensory system, motor coordination, or autonomic nervous system. Her gait was normal, and other physical examination findings were unremarkable.

Laboratory investigations revealed a white blood cell count of 8,580/μL, hemoglobin level of 14.1 g/dL, and hematocrit of 44.6%. The platelet count was 341×103/μL. Coagulation studies demonstrated an activated partial thromboplastin time (APTT) of 36.9 seconds, prothrombin time-international normalized ratio (PT-INR) of 1.06, fibrinogen level of 268 mg/dL, and D-dimer level <0.5 μg/mL. Inflammatory markers were within normal limits, with a C-reactive protein level of 0.04 mg/dL and an erythrocyte sedimentation rate of 6 mm/h. Thyroid function tests showed a free T3 level of 2.71 pg/mL, free T4 level of 1.68 ng/dL, and thyroid-stimulating hormone level of 0.43 μU/mL. Autoimmune test results were negative for anti-DNA, anti-SS-A, anti-SS-B, anti-neutrophil cytoplasmic, anti-thyroid peroxidase, anti-thyroglobulin, anti-acetylcholine receptor, anti-AQP4 (cell-based assay), and anti-myelin oligodendrocyte glycoprotein (MOG) (cell-based assay) antibodies. Test results for syphilis, human immunodeficiency virus, and human T-cell leukemia virus type 1 were also negative. Cerebrospinal fluid (CSF) analysis revealed an initial pressure of 150 mmH₂O (reference range: 60-200 mmH₂O). The CSF cell count was 5 cells/μL (100% mononuclear cells; reference range: 0-5 cells/μL). The protein concentration was 17 mg/dL (reference range: 15-50 mg/dL), and the glucose level was 56 mg/dL (reference range: 50-80 mg/dL). The immunoglobulin G index was 0.49 (reference range < 0.7), and the myelin basic protein level was < 31.2 pg/mL (reference range < 102 pg/mL). Oligoclonal bands were absent, and anti-MOG antibodies (cell-based assay) were not detected. Additionally, no cryptococcal or aspergillus antigens were detected. Polymerase chain reaction test results for herpes simplex and varicella-zoster viruses were negative. CSF cultures exhibited no bacterial growth.

**Figure 2:**
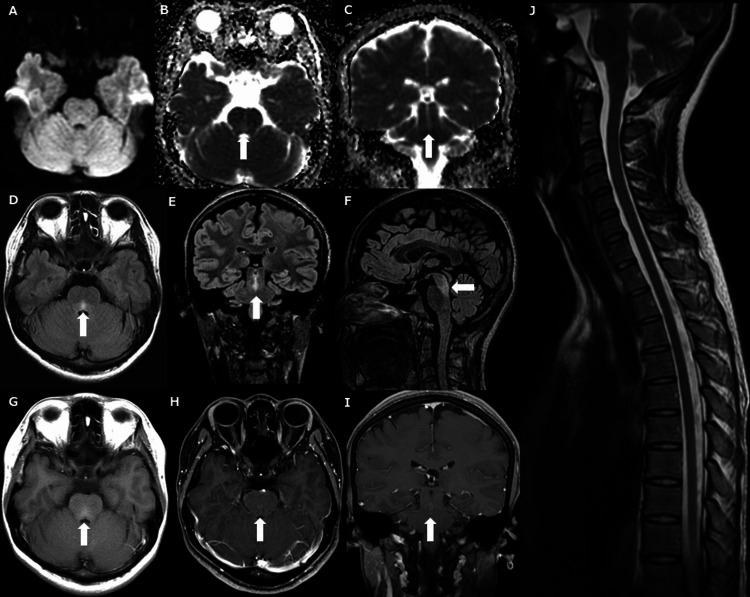
MRI at admission on day 61. Diffusion-weighted imaging (DWI) revealed no lesions (A). Apparent diffusion coefficient (ADC) map indicates a high-intensity lesion extending from the midbrain to the dorsal median pons (arrow) (B and C). Fluid-attenuated inversion recovery (FLAIR) imaging demonstrates a high-intensity lesion extending from the midbrain to the dorsal median pons (arrow) (D, E, and F). T1-weighted images display a high-intensity lesion extending from the midbrain to the dorsal median pons (arrow) (G). Contrast-enhanced T1-weighted images display focal enhancement (arrow) (H and I). Spinal cord MRI reveals no lesions (J). The lesion location can be anatomically explained as corresponding to the patient's diplopia. The presence of a hyperintense lesion on FLAIR with focal enhancement, in conjunction with the absence of signal change on DWI but high signal on ADC map, suggests an inflammatory etiology (brainstem encephalitis) based on imaging findings.

A detailed ophthalmologic examination revealed a corrected visual acuity of 20/20 bilaterally and intraocular pressures of 20.3 mmHg (reference range: 10-21 mmHg) in the right eye and 19.9 mmHg in the left eye. No orbital abnormalities were associated with the ocular pain, and no visual field defects were noted. The optic nerve papillae appeared normal, with no redness or swelling. Visual evoked potentials exhibited no abnormalities. Mild bilateral optic nerve atrophy secondary to elevated intraocular pressure was observed, and optical coherence tomography revealed retinal optic nerve fiber layer defects, consistent with open-angle preperimetric glaucoma. Brain magnetic resonance imaging (MRI) revealed a lesion extending from the midbrain to the dorsal median pons that was characterized by mild T1 hyperintensity, T2 fluid-attenuated inversion recovery (FLAIR) hyperintensity, and apparent diffusion coefficient (ADC) map hyperintensity (Figure 2). Magnetic resonance angiography indicated no abnormalities. Spinal cord MRI revealed no lesions (Figure 2). Whole-body computed tomography revealed no abnormalities.

**Figure 3:**
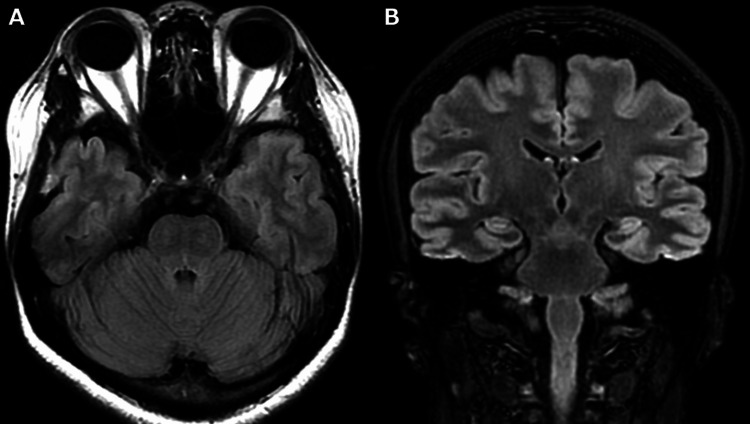
MRI on day 164. Fluid-attenuated inversion recovery (FLAIR) imaging following acute treatment indicates resolution of the lesion (A and B).

Acyclovir was administered until CSF polymerase chain reaction (PCR) results for herpes simplex virus (HSV) and varicella-zoster virus (VZV) were confirmed negative. Suspecting multiple sclerosis (MS) or neuromyelitis optica spectrum disorder (NMOSD), the patient received three courses of methylprednisolone pulse therapy (1000 mg/day for three days), leading to symptom improvement with only residual right-gaze discomfort. Oral prednisolone (0.5 mg/kg/day) was initiated. We considered the possibility of seronegative NMOSD, and tapered down the prednisolone dose and combined it with azathioprine (50 mg). MRI on day 164 revealed resolution of the lesions (Figure 3). In this case, the patient exhibited diplopia irrespective of the direction of gaze, and the brainstem lesion was considered the underlying cause of the symptoms. There was no imaging evidence of cavernous sinus syndrome, intracranial tumor, cerebral aneurysm, cerebral infarction, or cerebral hemorrhage that could have caused the ocular motility disorder. A comprehensive infectious workup was performed, including CSF PCR testing for HSV/VZV, as well as CSF and blood cultures. All results were negative, and the patient reported no history of recent infection or other vaccinations, except for the COVID-19 vaccination. MS was excluded, as the lesions were confined to the midbrain and dorsal median pons, did not exhibit temporal or spatial multiplicity, were oligoclonal bands-negative, and lacked lesions typical of MS, such as subcortical white matter and periventricular lesions [4]. The diagnosis of NMOSD, an autoimmune astrocytopathy targeting aquaporin-4 water channels, was excluded based on the 2015 International Consensus Diagnostic Criteria. The patient exhibited none of the core clinical characteristics, such as optic neuritis, acute myelitis, or area postrema syndrome. Furthermore, serum anti-AQP4 antibodies were negative on the cell-based assay. MOG antibody-associated disease (MOGAD), an inflammatory condition targeting myelin oligodendrocyte glycoprotein, was also ruled out, as anti-MOG antibodies were not detected [5]. Acute disseminated encephalomyelitis (ADEM) can present with brainstem lesions, but these are typically widely disseminated and bilateral [6] rather than localized, as in this case. There was no history of chronic alcoholism, malnutrition, or rapid correction of hyponatremia, which also ruled out osmotic demyelination syndrome [7]. Chronic lymphocytic inflammation with pontine perivascular enhancement responsive to steroids (CLIPPERS) is not a typical lesion observed on MRI, and this case did not meet the diagnostic criteria for probable CLIPPERS [8]. Posterior reversible encephalopathy syndrome (PRES) of the brainstem was ruled out in the absence of hypertension or renal dysfunction [9]. Although anti-ganglioside antibodies were not measured, the diagnosis of Bickerstaff brainstem encephalitis was excluded on the basis that there was no impaired consciousness or ataxia [10]. Behçet disease was excluded as there were no ocular lesions, oral or genital aphthae, skin lesions, or vascular symptoms [11]. Further, in this case, brainstem encephalitis occurred after vaccination with the COVID-19 vaccine and has not recurred since the onset of the disease, even after tapering the glucocorticoid dose. Based on these findings, brainstem encephalitis associated with COVID-19 vaccination was the most likely diagnosis in this case. To assess the relationship between vaccination and the neurological event, we applied the causality assessment criteria proposed by Butler et al. [12]. These criteria evaluate timing, alternative etiologies, and risk factors. In our case, symptom onset occurred within the typical six-week post-vaccination window, and extensive evaluation ruled out other infectious or autoimmune causes. Therefore, the case was classified as "probable." While the framework provides a basis for assessment, it does not constitute definitive biological proof, as no specific biomarkers or histopathological confirmation are currently available. Given the suspected vaccine-associated etiology, azathioprine was discontinued, and prednisolone was gradually tapered. Residual discomfort persisted at maximal right lateral gaze; however, no symptom recurrence or new MRI lesions were observed at day 164.

## Discussion

In addition to the COVID-19 vaccine, various vaccines and adjuvants can induce autoimmune reactions [13]. Although rare, reports of brainstem encephalitis following vaccination against influenza exist, with the mechanism thought to be similar to that of ADEM, in which inflammation around small blood vessels is the main pathological feature [14]. The possible mechanism of encephalitis after vaccination against COVID-19 has been proposed to involve vaccine-induced inflammatory cytokines and molecular mimicry between the SARS-CoV-2 spike protein and myelin basic protein. Classification as an immune-mediated disease is supported by the success of immunosuppressive therapy [15]. mRNA vaccines stimulate both acquired and innate immunity through a mechanism different from that of conventional vaccines, with the mRNA encoding the protein encapsulated in lipid nanoparticles and delivered to cells, where it is translocated into the cytoplasm and exerts its function. Neurological complications associated with COVID-19 vaccines may be related to acute inflammation induced by the SARS-CoV-2 spike protein and vascular endothelial dysfunction caused by destabilization of the renin-angiotensin system due to the interaction of angiotensin converting enzyme 2 with the spike proteins, subunits, and peptide fragments. Other possibilities include activation of toll-like receptors by ionized lipids in lipid nanoparticles, potentially triggering an inflammatory response [16]. In this case, brainstem encephalitis was also suspected to be an immune-mediated reaction caused by vaccination; however, no evidence of a causal relationship with the vaccination was identified.

Inflammatory demyelinating diseases of the central nervous system can also involve the brainstem. In MOGAD, lesions more frequently involve the middle cerebellar peduncle, whereas AQP4 antibody-positive NMOSD more commonly affects the area postrema, reflecting antigen-specific predilection sites of injury. Additionally, reports of AQP4 antibody-positive NMOSD with pathogenic autoantibodies suggest that the periventricular area, including the area postrema, may lack an adequate blood-brain barrier and serve as a site where IgG can easily enter the central nervous system [17]. Consistent with these mechanisms, periventricular lesions observed in this case may reflect an immune-mediated reaction affecting a vulnerable region of the blood-brain barrier or corresponding to the site of antigen expression related to the post-vaccination immune response.

Brainstem encephalitis following COVID-19 vaccination is extremely rare. However, the clinical course in this case, characterized by fluctuations between improvement and deterioration, resembled that of a previously reported case [18]. In the previously reported case, diplopia developed acutely after the first COVID-19 vaccination, gradually worsened, partially improved, and then worsened again, fluctuating without complete resolution. The diplopia worsened three weeks after the first vaccination and again the day after the second vaccination. The patient exhibited bilateral abduction deficits, and MRI revealed a lesion in the dorsal median pons. The patient was treated with methylprednisolone pulse therapy, followed by oral glucocorticoids, and her symptoms improved without recurrence [18]. Key differences between the present case and the previous report include the timing of symptom onset. In the previous case, symptoms began the day after the first vaccination, while in the present case, symptoms began a few days after the second vaccination. Furthermore, in the previous case, symptoms resolved completely, whereas in the present case, only discomfort in the right visual pole persisted. Although the mechanism of paroxysmal symptoms in MS remains unclear, it has been suggested that partially demyelinated axons may cause paroxysmal symptoms through lateral spreading conduction and that horizontal paroxysmal diplopia may result from stimulation of the medial longitudinal fasciculus by lesions in the midbrain and dorsal median pons [19]. In the present case, the patient may have experienced variable paroxysmal symptoms due to a mechanism similar to MS. However, the mechanism could also be transient edema, similar to PRES, that can occur after vaccination. The immune response leading to systemic inflammation, vascular endothelial dysfunction induced by the COVID-19 mRNA vaccine, and damage to vascular endothelial cells by spike proteins has been proposed to contribute to PRES [20]. Although the clinical presentations in this case were not typical of PRES, a similar pathophysiological mechanism involving transient edema cannot be excluded, particularly given the hyperintense lesions observed on FLAIR imaging and ADC mapping.

This case is similar to a previous case in which the patient developed transient, reproducible diplopia after vaccination, with repeated improvements and worsening, developed a lesion in the dorsal median pons, and responded well to glucocorticoid treatment (Table 1) [18]. However, there have been only a few verified cases, and further case accumulation and investigation are necessary.

**Table 1:** Comparison of clinical characteristics between the present case and a previously reported case of post-vaccination brainstem encephalitis.

Feature	Present case	Previous case (Kobayashi et al. [18])
Vaccine type	BNT162b2 (mRNA)	BNT162b2 (mRNA)
Onset after vaccination	A few days after the second dose	1 day after the first dose
Oculomotor deficits	Left: Adduction/infraduction restriction; right: abduction restriction, with pain	Bilateral abduction restriction
MRI findings	Lesion extending from the midbrain to the dorsal median pons	Lesion in the dorsal pons
Treatment	Methylprednisolone pulse + oral prednisolone + azathioprine	Methylprednisolone pulse + oral prednisolone
Clinical outcome	Improvement with residual discomfort	Improvement

A major strength of this report is the detailed documentation of a rare presentation of isolated brainstem encephalitis. However, the principal limitation is that this is a single case report (n = 1), which prevents definitive establishment of causality. In addition, the absence of specific biomarkers for vaccine-associated encephalitis further complicates etiological confirmation.

## Conclusions

We report a case of brainstem encephalitis following COVID-19 vaccination. Based on the temporal association and the exclusion of alternative infectious and autoimmune etiologies, this case most likely represents a brainstem encephalitis probably associated with COVID-19 vaccination. Nevertheless, given the inherent limitations of a single case report, these findings should be interpreted with caution. Future research should focus on a large-scale study to establish the true incidence, potential risk factors, and underlying immunopathological mechanisms of post-vaccination brainstem syndromes, allowing for more robust safety monitoring.

Given that the benefits of vaccination against COVID-19 are substantial, particularly in high-risk situations, we do not recommend withholding vaccination. However, it is important to recognize that brainstem encephalitis, although rare, may occur after COVID-19 vaccination.
